# Prevalence of diabetes mellitus among tuberculosis patients in Sub-Saharan Africa: a systematic review and meta-analysis of observational studies

**DOI:** 10.1186/s12879-019-3892-8

**Published:** 2019-03-13

**Authors:** Animut Alebel, Amsalu Taye Wondemagegn, Cheru Tesema, Getiye Dejenu Kibret, Fasil Wagnew, Pammla Petrucka, Amit Arora, Amare Demsie Ayele, Mulunesh Alemayehu, Setegn Eshetie

**Affiliations:** 1 College of Health Sciences, Debre Markos University, P.O. Box 269, Debre Markos, Ethiopia; 2 College of Nursing, University of Saskatchewan, Saskatoon, Canada; 3 School of Life Sciences and Bioengineering, Nelson Mandela African Institute of Science and Technology, Arusha, Tanzania; 4 School of Science and Health, Western Sydney University, Penrith, NSW 2751 Australia; 5 Translational Health Research Institute, Western Sydney University, Penrith, NSW 2751 Australia; 6 Discipline of Child and Adolescent Health, Sydney Medical School, Faculty of Medicine and Health, The University of Sydney, Westmead, NSW 2145 Australia; 7 Oral Health Services, Sydney Local Health District and Sydney Dental Hospital, NSW Health, Surry Hills, NSW 2010 Australia; 8 College of Medicine and Health Sciences, University of Gondar, Gondar, Ethiopia

**Keywords:** Type1/type 2 diabetes mellitus, Tuberculosis, Sub-Saharan Africa

## Abstract

**Background:**

Tuberculosis and diabetes mellitus are significant global public health challenges. In Sub-Saharan Africa, study findings regarding prevalence of diabetes mellitus amongst tuberculosis patients have been inconsistent and highly variable. Therefore, this systematic review and meta-analysis estimates the overall prevalence of diabetes mellitus among tuberculosis patients in Sub-Saharan Africa.

**Methods:**

Four international databases (PubMed, Google Scholar, Science Direct and Cochrane Library) were systematically searched. We included all observational studies reporting the prevalence of DM among TB patients in Sub-Saharan Africa. All necessary data for this review were extracted using a standardized data extraction format by two authors (CT and AA1). STATA Version 14 statistical software was employed to conduct meta-analysis. The Cochrane Q test statistics and *I*^*2*^ test were used to assess the heterogeneity of the studies. Finally, a random effects meta-analysis model was computed to estimate the pooled prevalence of diabetes mellitus in TB patients. Besides, subgroup analysis was done based on different factors.

**Results:**

In the meta-analysis, sixteen studies fulfilled the inclusion criteria and were included. The findings of these 16 studies revealed that the pooled prevalence of diabetes mellitus among tuberculosis patients in Sub-Saharan Africa was 9.0% (95% CI: 6.0, 12.0%). The highest prevalence of diabetes mellitus among tuberculosis patients was found in Nigeria (15%), followed by Tanzania (11%), and then Ethiopia (10%). Besides, the prevalence of diabetes mellitus among HIV infected TB patients was (8.9%) which is slightly higher than HIV uninfected (7.7%) TB patients.

**Conclusion:**

Diabetes mellitus among tuberculosis patients in Sub-Saharan Africa was significantly high. Moreover, this study found that there was a high prevalence of DM among HIV infected than uninfected TB patients. It is strongly recommended to screen for DM among TB patients and special emphasis should be given for early screening of DM among TB/HIV co-infected patients.

**Electronic supplementary material:**

The online version of this article (10.1186/s12879-019-3892-8) contains supplementary material, which is available to authorized users.

## Background

Currently, non-communicable diseases (NCDs) are a growing worldwide epidemic that disproportionately affects low- and middle-income countries (LMIC) where, concomitantly, the burden of infectious diseases is high. The prevalence of NCDs in low-income countries in 1990 was reported to be 47%, but it is projected to rise to 69% by 2020 and NCDs will likely exceed cases of communicable diseases by 2030 [1]. Advancing industrialization and urbanization have contributed to lifestyle changes, primarily in dietary habits, leading to increased rates of obesity and Type II diabetes mellitus (DM). Globally, there are approximately 422 million adults living with DM of which about 80% of cases reside in LMIC [2–4], where concomitantly communicable diseases, such as tuberculosis (TB), are often endemic [5]. Type 2 DM accounts for about 90% of the diabetes with even higher prevalence in urban and aged populations [6].

The dual burden of communicable and non-communicable epidemics facing Sub-Saharan Africa (SSA) further complicates the experiences and implications of these diseases. There are known negative impacts in co-morbid cases [7]. Some studies showed that DM and TB are the two interlaced diseases [8, 9]. This strong correlation is especially, accentuated in LMIC, where almost 95% of the world’s population with TB and 70% with DM live [10]. Different studies conducted elsewhere disclosed that presence of DM increases the life time risk of developing TB by three-folds [8, 11, 12]. The physiologic association between the two diseases is not fully explored, but studies suggested that DM weakened the immune response, which, in turn, enhances the infection of *Mycobacterium tuberculosis* and/or progression from latent to active disease state [13]. Alternately, TB can temporarily cause impaired glucose tolerance and might predispose patients to DM [14]. Moreover, chronic infections such as TB are associated with idiopathic hyperglycemia, which occurs due to increased production of counter-regulatory stress hormones such as epinephrine, glucagon, cortisol, and growth hormone which act synergistically [15].

In SSA, study findings regarding the prevalence of DM among TB patients differ by geographical region and the background characteristics of the study participants [9, 16–30]. These studies reported that the prevalence of DM among TB patients in SSA ranged from 1.9% in Benin [30] to 38% in Nigeria [22]; however, in SSA there was no regional-based study, which considers the prevalence of DM among TB patients. Therefore, the aim of this systematic review and meta-analysis was to estimate the pooled prevalence of DM among TB patients in SSA. The findings of this systematic review and meta-analysis will highlight the prevalence of DM among TB patients in SSA with implications to improve health care workers’ interventions, to ensure their cost-effectiveness, and accelerate the reduction of the DM prevalence among TB patients.

## Methods

This systematic review and meta-analysis was intended to estimate the pooled prevalence of DM among TB patients in SSA. The protocol for this review was registered in the International Prospective Register of Systematic Reviews (PROSPERO), University of York Centre for Reviews and Dissemination (Registration Number CRD42017073403) on the 31th of August, 2017. To ensure scientific rigor, the Preferred Reporting Items for Systematic Reviews and Meta-Analysis (PRISMA) guidelines was used [31]. Four international databases-PubMed, Google Scholar, Science Direct and The Cochrane Library, were systematically searched. To search relevant articles for this study, we used the following keywords “prevalence”, “diabetes mellitus”, “type one diabetes mellitus”, “type two diabetes mellitus”, “tuberculosis”, and “Sub-Saharan Africa”. The key terms were used separately and/ or in combination using Boolean operators like “OR” or “AND”. The literature search from the above databases was done from August 10 to September 9, 2017. All papers published until the 9th of September, 2017 were included in this review.

### Eligibility criteria

#### Inclusion criteria

##### Study area

All studies conducted in SSA countries

##### Publication condition

Manuscripts published in peer reviewed journals

##### Study design

For this review, we included all observational study designs (cross-sectional, case-control, and cohort studies) reporting the prevalence of DM among TB patients in SSA.

##### Language

Articles reported in the English language were included.

#### Exclusion criteria

We excluded papers that were not fully accessible, after at least two email contact attempts with the primary authors. Exclusion of these articles was due to inability to assess the quality of articles in the absence of full text.

### Outcome of interest

Primarily, this study aims to estimate the pooled prevalence of DM among TB patients in SSA. The prevalence was calculated by dividing the number of individuals who have DM to the total number of patients who have TB (sample size) multiplied by 100. Secondly, this study aims to compare the prevalence of DM among HIV infected and uninfected TB patients.

### Operational definitions

Based on the World Health Organization (WHO) DM diagnostic criteria, the patent is considered as diabetic if he or she fulfilled the following two conditions: when the random blood glucose (RBG) value is ≥200 mg/dl and/or fasting blood glucose (FBG) value ≥126 mg/dl on two separate occasions [32] plus the patient shows the classical signs and symptoms of DM.

Positive TB status (PTB) was diagnosed when a patient fulfilled at least two of the following criteria: positive sputum smear by microscopic examination of Ziehl-Neelsen-stained sputum slides for acid-fast bacilli, chest radiographs with suggestive features of TB, and/or clinical symptoms and signs of TB [33].

### Data extraction

Two authors (CT and AA1) independently extracted all necessary data using a standardized data extraction format. The data extraction format included primary author, publication year, country of the study, study design, sample size, and prevalence.

### Quality assessment

First of all, the Newcastle-Ottawa Scale (NOS) quality assessment tool for cross-sectional studies was adapted [34]. Then after, the two authors (AA1 and CT) independently assessed the quality of included peer reviewed articles using the above tool. If there were differences in the scoring of articles between the two reviewers, the differences were addressed by taking the mean score of the two authors or by involving the third author. After reviewing different literatures, we declared that articles scored ≥6 points out of 10 were considered to be high-quality (see Additional file 1). The NOS tool emphasized on three main issues. The principal component of the tool graded from five stares and mainly emphasized on the methodological quality of each primary study. The other component of the tool graded from two stars and mainly concerns about the comparability of each study. The last component of the tool graded from three stars and used to assess the outcomes and statistical analysis of each original study.

### Statistical analysis, sub-group analysis, and publication bias

We used a Microsoft Excel spreadsheet for data extraction and STATA Version 14 statistical software for data analysis. The descriptive data were presented using a table to describe the characteristics of each primary study. Besides, the point prevalence of each study as well as the overall prevalence were described using a forest plot graph. The forest plot was interpreted as follows: the horizontal line shows the 95%CI and the black box represents the Wight of each study. Moreover, an explanatory data analysis using Q-statistics and *I*^2^ test was conducted to assesses the random variations between each primary study [35]. In this study, heterogeneity was interpreted as an I^2^ value = 0% no heterogeneity, 25% = low, 50% = moderate, and 75% = high [36]. Based on the above testes, the primary studies included in this meta-analysis exhibited a significant random variation (*I*^*2*^ = 97.5% with Cochrane Q-statistics *p*-value < 0.001), which forced us to use a random effects meta-analysis model to compute the Der Simonian and Laird’s pooled effect. Additionally, we computed a subgroup analysis based on different variables including country of the primary studies, HIV infection status, and sample size. Besides, an advanced statically analysis like a univariate meta-regression model was done based on sample size and year of publication as covariates to identify the sources of random variations among included primary studies. Lastly, publication bias was assessed using Egger’s and Begg’s tests at 5% level of significant [37].

## Results

**Fig. 1:**
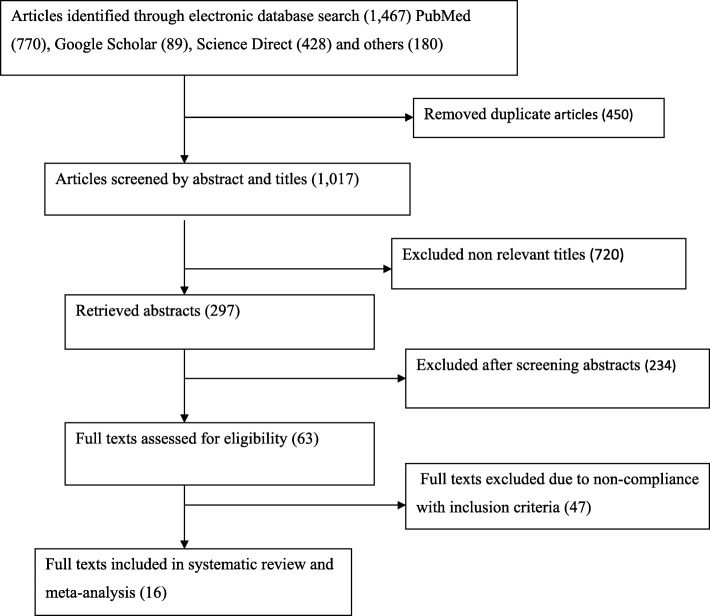
Flow chart of study selection for systematic review and meta-analysis of the prevalence of DM among TB patients in SSA

In the first step of our search, 1467 articles were retrieved on the prevalence of DM among TB patients. Of these, 450 articles were excluded due to duplication. From the remaining 1017 articles, 720 articles were excluded after reviewing of their titles and abstracts based on an assessment as they were non-relevant to the aim of this review. The remaining 297 abstracts were screened, yielding an additional 234 being excluded as non-relevant to this study. A total of 63 full text articles were accessed and assessed for eligibility based on the pre-set inclusion criteria. This step resulted in further exclusion of 47 articles primarily due to the study locations. Among these, five of the studies were conducted in Oceania regions [38–42], seven from North America [11, 43–48], three from South America, two from Europe [49–51], and 30 from Asia [17, 52–81] (Fig. 1). As a result, 16 studies met the eligibility criteria and were included in the systematic review and meta-analysis.

### Characteristics of original studies

As described in Table 1, the 16 studies were published between 1999 to 2017. In the current meta-analysis, 13,286 study participants were included to estimate the pooled prevalence of DM among TB patients. Regarding study design, more than half (56.3%) of the studies are cross-sectional. The sample size of the studies ranged from 107 to 4000. The lowest prevalence (1.9%) of DM was reported in a study conducted in Benin [30], whereas the highest prevalence (38%) was reported in a study conducted in Nigeria [22]. In this meta-analysis, nine SSA countries were represented. Three of the studies were from Ethiopia [16, 26, 28]; four from Nigeria [19–22], two from Tanzania [9, 23], one each from Guinea-Bissau [25], Cotonou-Benin [30], Uganda [24], Guinea [29], Madagascar [17], Kenya [18], and Cameroon [27].

**Table 1:** Descriptive summary of 16 studies included in the meta-analysis of the prevalence of diabetes mellitus among Tuberculosis patients in Sub-Saharan Countries, 2017

Study period	Publication year	Country	Study design	Study period	Sample size	Prevalence with 95% CI
Ade et al. [30]	2015	Cotonou-Benin	Cross-sectional	June–July/ 2014	159	1.9 (1, 5)
Faurholt-Jepsen et al. [9]	2011	Tanzania	Case control	Apr 2006-Jan 2009	803	16 (14, 19)
Haraldsdottir et al. [25]	2015	Guinea-Bissau	NR	July 2010–July 2011	107	2.8 (1, 8)
Kibirige et al. [24]	2013	Uganda	Cross-sectional	Sep 2011-Feb 2012	260	8.5 (6, 12)
Ogbera et al. [21]	2014	Nigeria	Cross-sectional	Sep 2010–Mar 2012	3, 376	4.8 (4, 8)
Olayinka et al. [19]	2013	Nigeria	Cross-sectional	NR	351	5.7 (4, 9)
Workneh et al. [16]	2016	Ethiopia	Cross-sectional	Sep 2103–Sep 2014	1, 314	8.3 (7, 10)
Ogbera et al. [20]	2015	Nigeria	Descriptive observational study	Mar 2011-July 2012	4, 000	12 (11, 13)
Getachew et al. [26]	2014	Ethiopia	Cross-sectional	Oct 2011-Aug 2012	199	8.5 (5, 13)
Damtew et al. [28]	2014	Ethiopia	Cross-sectional	Feb2014-May 2014	120	16 (10, 23)
Balad et al. [29]	2006	Guinea	NR	Feb -June 2002	388	3.4 (2, 6)
Rakotonirina et al. [17]	2014	Madagascar	Descriptive	July15-Oct.30,2013	156	5.8 (3, 11)
Mugusi et al. [23]	1999	Tanzania	NR	NR	506	6.7 (5, 9)
Owiti et al. [18]	2017	Kenya	Cross-sectional	Jan -June 2016	454	6.7 (3, 7)
Fonkeng et al. [27]	2017	Cameroon	Cross-sectional	Nov 2014-July 2015	222	9.5 (6, 19)
Ekeke et al. [22]	2017	Nigeria	Prospective study	NR	871	38 (35, 41)

### Quality assessment

The quality score of each original study ranged from four to eight (see Additional file 1). Regarding the sampling techniques used, majority (*n* = 15, 93.8%) of the included studies used consecutive sampling technique to select study participants [9, 16, 17, 20, 21, 23–25, 27–30, 82]. Concerning the laboratory methods used to diagnose DM, seven studies used FBG [17, 19, 21, 28–30], four used RBS [16, 24, 25, 27], one each used OGT [23], HbA1c [18], and FBG and OGT [9], two studies did not report the methods used [20, 82]. Regarding study settings, seven out of sixteen studies conducted in hospitals [19, 23, 24, 26–29] and five in health centers [16, 18, 20, 30, 82].

### Meta-analysis

**Fig. 2:**
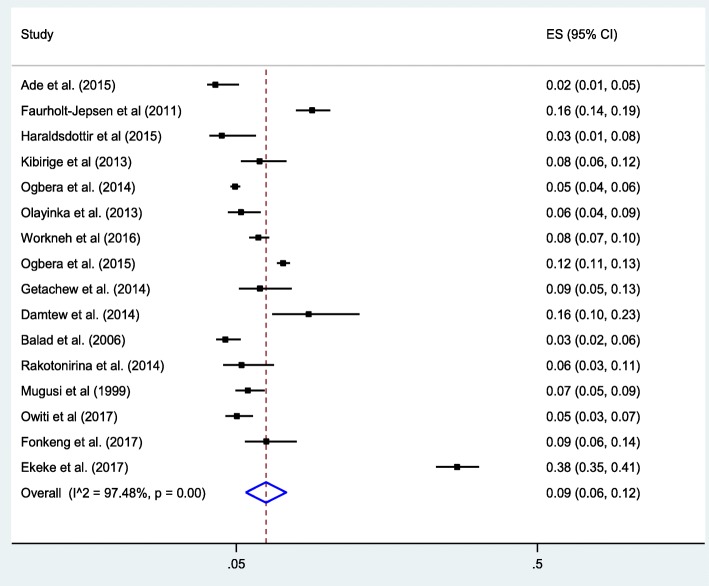
Forest plot of the pooled prevalence of DM among TB patients in SSA

As presented in Fig. 2, this meta-analysis found that that the pooled proportion of DM among TB patients in SSA was found to 9% (95% CI: 6, 12). The included studies exhibited high heterogeneity (*I*^*2*^ = 97.5% with Cochrane Q-statistics *p*-value < 0.001) because of this, the final overall prevalence was computed based on a random effects meta-analysis model. Additionally, we conducted an advanced statistical meta-analysis model such as a univariate meta-regression model by considering publication year and sample size as covariates to identify the possible sources of random variations across primary studies. Nevertheless, these variables were not statistically significant source of heterogeneity (Table 2). Finally, the possibility of publication biases across primary studies were examined using Begg’s correlation and Egger’s regression tests. The test results, showed that there was no statistically significant publication bias across the included studies (*p*-values = 0.15 and = 0.3 respectively).

**Table 2:** Related factors with heterogeneity of diabetes mellitus prevalence among tuberculosis patients in the current meta-analysis (based on univariate meta-regression)

Variables	Coefficient	P-value
Publication year	− 0.083	0.79
Sample size	0.001	0.47

### Subgroup analysis

In this meta-analysis, we performed a subgroup analysis based on the country where the studies were conducted and sample size of the studies. Accordingly, the highest prevalence was observed in Nigeria with a prevalence of 15% (95% CI: 7, 23), followed by Tanzania 11% (95% CI: 9, 12), and then Ethiopia at 10% (95% CI: 6, 13) (Table 3). With regard to sample size, the prevalence of diabetes was higher in studies having a sample size ≥ 300 patients, 11% (95% CI: 7, 15) compared to those having a sample size < 300 patients, 7% (95% CI: 4, 10). We compared the prevalence of DM among HIV infected and uninfected TB patients by including the reports of eight studies [16, 18, 22, 24, 26–28, 82]. The results of these studies indicated that the prevalence of DM among HIV infected TB patients was 8.9% (95CI 6.5, 11.3) which is higher than uninfected TB patients estimated at 7.7% (95%CI: 5.4, 10.1). High heterogeneity (I^2^ = 78.3% and *P*-value < 0.001) was observed across the included studies; hence, a random effects meta-analysis model was employed to compare the prevalence of DM between HIV infected and uninfected TB patients. Furthermore, we conducted a subgroup analysis based on the geographic residence (urban versus rural) of patients. However, in this study, there was no difference in the prevalence of DM among TB patients between urban (9%) and rural (9%) (Table 3).

**Table 3:** Subgroup prevalence of diabetes mellitus among tuberculosis patients in Sub-Saharan African countries, 2017 (*n* = 16)

Variables	Characteristics	Number of studies included	Sample size	Estimate (95% CI)
Country	Nigeria	4	4998	15 (7, 23)
	Ethiopia	3	1633	10 (6, 13)
	Tanzania	2	1309	11 (9, 12)
	Others	7	5345	5 (3, 7)
Sample size	≥300	9	12,063	11 (7, 15)
	< 300	7	1, 223	7 (4, 10)
HIV infection	HIV Positive	9	1365	8.9 (6.5, 11.3)
	HIV negative	9	6, 584	7.7 (5.4, 10.1)
Residence	Urban	5	2, 269	9 (8, 11)
	Rural	5	1700	9 (5, 12)
Overall		16	13,286	9 (6, 12)

## Discussion

To the best our knowledge, this meta-analysis is the first of its kind to estimate the pooled prevalence of DM among TB patients in SSA region. However, the increased prevalence of DM-TB co-morbidity is rapidly becoming a major public health problem throughout the world, including resource-limiting settings, especially in high TB burden countries. Determining the pooled prevalence of DM among TB patients potentially catalyzes program and policy-makers to take remedial action.

The findings of this study showed that the pooled prevalence of DM among TB patients was 9% (95% CI: 6, 12). The prevalence obtained from this meta-analysis is in line with the estimated prevalence of DM (8.5–16.4%) among TB patients in SSA [19, 29, 83–85], and South America 11.1% (IQR: 6.1–14%) [86] as reported in a previous systematic review conducted on DM and TB co-morbidity. However, this finding is higher than the estimated prevalence of DM (2.1–6.7%) among the general SSA population [87]. This finding is also slightly higher than the estimated prevalence of DM (5.9%) among TB patients in European countries [86]. On the other hand, our finding is much lower than the estimated prevalence of DM among TB patients in Asian countries 17% (IQR 11.4–25.8%), North America 23.6% (IQR: 17.3–35.4%), and Oceania 23.3% (IQR: 12.8–39.0%) as reported in a previous systematic review conducted on DM and TB co-morbidity [86]. A possible source of regional variation in the prevalence of DM among TB patients might be attributable to the differences in the general population prevalence of DM in the respective countries. The above discrepancies align with the estimated prevalence of DM among the adult population [88]. According to the WHO (2016), the prevalence of DM by region were 7.1% (SSA), 8.3% (America), 13.7% (Mediterranean), 7.3% (Europe), 8.6% (South-East Asia), and 8.4% (Western Pacific) [88].

In this meta-analysis, the lowest prevalence (1.9%) of DM was observed in a study conducted in Benin [30], whereas the highest prevalence (38%) was observed in a study conducted in Nigeria [22]. This high disparity in the prevalence of above studies could be due to the various techniques used to diagnose DM among TB patients and possibly the effects of co-morbidities such as HIV. From the included 16 studies, seven studies used FBG technique to diagnose DM [17, 19, 21, 28–30]. Concerning the diagnosis methods of DM among TB patients, no standard diagnostic method has been advocated for TB patients; hence, either a RBS, FBG, OGTT or HbA1c test can be used alone or in combination [7].

The subgroup analysis of this study showed that the pooled prevalence of DM among TB patients in Nigeria was 15% (95% CI: 7, 23) which is higher than the prevalence in Tanzania 11% (95% CI: 9, 12), Ethiopia 10% (95% CI; 6, 13), and others 5% (95% CI: 3, 7). The possible explanations for this variation might be due to socioeconomic and sociocultural differences between the populations. Another possible explanation for this variation might be differences in the screening methods used, and variations in the prevalence of DM in the general population of the respective countries. Regional variation with regard to the burden of TB might have an impact on the prevalence of DM. According to WHO (2016), the incidence of TB in Nigeria was 322 per 100,000 while lower incidence of TB was noted in other countries like Ethiopia and Tanzania [89]. Similarly, from this subgroup analysis, we observed that the estimated prevalence of DM among TB patients in Nigeria was 15%, which is much higher than the estimated prevalence of DM among the general population. Likewise, from the above subgroup analysis, we found that the pooled prevalence of DM among TB patients in Ethiopia was 10%, which is almost twice the prevalence of DM among the general population. Moreover, the estimated prevalence of DM among TB patients in Tanzania was 11%, which is also higher than the estimated prevalence of DM among the general population.

In this study, we tried to compare the prevalence of DM between HIV infected and uninfected TB patients. This result reflects that the overall prevalence of DM among HIV infected TB patients is (8.9%) which is lightly higher than uninfected TB patients (7.7%). This finding is comparable with previous findings showing that people living with HIV have a higher risk of developing DM due to side effects of certain HIV medicines which may increase blood glucose levels and lead to Type 2 DM [90]. Studies have suggested that prolonged exposure of the anti-retroviral medication can be an aggravating factor for the occurrence of DM. In addition, another study also indicated that HIV infection was significantly associated with higher incidence of DM, with a reported incidence of DM per 100 person-years among HIV infected group as 2.44, which is significantly higher than the incidence reported in their HIV negative counterparts (1.89 per 100 person-years) [91].

### Limitations of the study

Despite the authors performed a comprehensive search using different databases to address all articles conducted on the prevalence of DM among TB patients in SSA, this systematic review failed to include papers published other than the English language. To boot, this systematic review included only 16 studies involving 13,286 TB patients. Therefore, this systematic review relatively analyzed data of limited number of study participants and this factor could significantly affects the estimated reports. Furthermore, during our search, we found studies only from the nine countries of SSA region and other countries may be under-represented due to the limited number of studies included. At last, the results obtained from this review should be interpreted cautiously because more than half (56.3%) of the studies included in the meat-analysis had cross-sectional study design. It is well known that the reports of cross-sectional type of study designs are highly influenced by confounding variables.

## Conclusion

In conclusion, the pooled prevalence of DM among TB patients in SSA was significantly high. This review found that the prevalence of DM among HIV-infected TB patients is higher than HIV-uninfected TB patients. Because of the frequent co-morbidity of these two diseases, focusing on signs of diabetes among patients with TB, particularly if the risk factors are present, could contribute to improved detection and early treatment of diabetes in this population. Therefore, based on our findings, we recommend consideration of the potential for routine screening for DM among TB patients. Moreover, a special emphasis should be given for early screening of DM among TB/HIV co-infected patients.

## Additional file

Additional file 1: Quality score of each study. (DOCX 27 kb)

## Abbreviations

CI: Confidence Interval
DM: Diabetes Mellitus
FBG: Fast Blood Glucose
HIV: Human Immunodeficiency Virus
NCD: Non-communicable Disease
NOS: Newcastle Ottawa Scale
PTB: Pulmonary Tuberculosis
RBG: Random Blood Glucose
SSA: Sub-Saharan Africa
TB: Tuberculosis
