# Supplementary material for: Prevalence of diabetes mellitus among tuberculosis patients in Sub-Saharan Africa: a systematic review and meta-analysis of observational studies
Source: BMC Infect Dis. 2019 Mar 13;19:254. doi: 10.1186/s12879-019-3892-8 (PMC6417234; doi:10.1186/s12879-019-3892-8)
Supplement: Supplementary file 1 — Quality score of each study. (DOCX 27 kb) [file 12879_2019_3892_MOESM1_ESM.docx]

Additional file 1

| **Table 4. Shows the quality score of each study using** Newcastle-Ottawa Scale **(NOS) quality assessment tool adapted for cross-sectional studies**. | | | | | | | | |
| --- | --- | --- | --- | --- | --- | --- | --- | --- |
| **Sample selection (maximum 5 stars)** | Ade et al.[[30](#_ENREF_30)] | Faurholt-Jepsen et al.[[9](#_ENREF_9)] | Haraldsdottir et al.[[25](#_ENREF_25)] | Kibirige et al.[[24](#_ENREF_24)] | Ogbera et al.[[21](#_ENREF_21)] | Olayinka et al. [[19](#_ENREF_19)] | Workneh et al.[[16](#_ENREF_16)] | Ogbera et al. [[20](#_ENREF_20)] |
| 1.**Representativeness of the sample**: Truly representative of the average in the target population. * (all subjects or random sampling), b) Somewhat representative of the average in the target population. * (non-random sampling), c) Selected group of users and d) No description of the sampling strategy | d | a* | a* | a* | a* | a* | a* | a* |
| 2. **Sample size**: a) **Justified and satisfactory.** * and b) Not justified. | * | * | * | * | * | * | * | b |
| 3. **Non-respondents**: a) Comparability between respondents and non-respondents characteristics is established, and the ***response rate is satisfactory.*** *, b) The response rate is unsatisfactory, or the comparability between respondents and non-respondents is unsatisfactory and c) No description of the response rate or the characteristics of the responders and the non-responders. | c | a* | c | c | c | c | C | c |
| 4) **Ascertainment of the exposure (risk factor)**: a) Validated measurement tool. **, b) Non-validated measurement tool, but the tool is available or described. *, c) No description of the measurement tool. | b | c | b | b | a | b | B | b |
| **Comparability: (Maximum 2 stars)** |  |  |  |  |  |  |  |  |
| 1) The subjects in different outcome groups are comparable, based on the study design or analysis. Confounding factors are controlled. a) The study controls for the most important factor (select one). * b) The study control for any additional factor. * | b* | b* | b* | b* | b* | b* | b* | a* |
| **Outcome: (Maximum 3 stars)** |  |  |  |  |  |  |  |  |
| 1) Assessment of the outcome: a) Independent blind assessment. **, b) Record linkage. ** c) Self report. * and d) No description. | a** | a** | a** | a** | a** | a** | a** | a** |
| 2) Statistical test: a) The statistical test used to analyze the data is clearly described and appropriate, and the measurement of the association is presented, including confidence intervals and the probability level (p value). * and b) The statistical test is not appropriate, not described or incomplete. | * | * | * | * | * | * | * | * |
| **Overall quality score (máximum of eight stars)** | 6 | 8 | 7 | 7 | 8 | 6 | 8 | 6 |
| **Sample selection (maximum 5 stars)** | Getachew et al.[[26](#_ENREF_26)] | Damtew et al. [[28](#_ENREF_28)] | Balad et al. [[29](#_ENREF_29)] | Rakotonirina et al.[[17](#_ENREF_17)] | Mugusi et al. [[23](#_ENREF_23)] | Owiti et al. [[18](#_ENREF_18)] | Fonkeng et al.[[27](#_ENREF_27)] | Ekeke et al.[[22](#_ENREF_22)] |
| 1.Representativeness of the sample: Truly representative of the average in the target population. * (all subjects or random sampling), b) Somewhat representative of the average in the target population. * (non-random sampling), c) Selected group of users and d) No description of the sampling strategy | a* | a* | d | b* | a* | d | a* | a* |
| 2. Sample size: a) **Justified and satisfactory.** * and b) Not justified. | b | b | * | b | b | * | * | * |
| 3. Non-respondents: a) Comparability between respondents and non-respondents characteristics is established, and the ***response rate is satisfactory.*** *, b) The response rate is unsatisfactory, or the comparability between respondents and non-respondents is unsatisfactory and c) No description of the response rate or the characteristics of the responders and the non-responders. | c | c | a* | c | c | c | C | c |
| 4) Ascertainment of the exposure (risk factor): a) Validated measurement tool. **, b) Non-validated measurement tool, but the tool is available or described. *, c) No description of the measurement tool. | b | b | b | b | b | b | C | b |
| **Comparability: (Maximum 2 stars)** |  |  |  |  |  |  |  |  |
| 1) The subjects in different outcome groups are comparable, based on the study design or analysis. Confounding factors are controlled. a) The study controls for the most important factor (select one). * b) The study control for any additional factor. * | a* | a* | b* | a* | b* | b* | b* | b* |
| **Outcome: (Maximum 3 stars)** |  |  |  |  |  |  |  |  |
| 1) Assessment of the outcome: a) Independent blind assessment. **, b) Record linkage. ** c) Self report. * and d) No description. | a** | a** | a** | a** | d | a** | a** | a** |
| 2) Statistical test: a) The statistical test used to analyze the data is clearly described and appropriate, and the measurement of the association is presented, including confidence intervals and the probability level (p value). * and b) The statistical test is not appropriate, not described or incomplete. | b | * | b | b | * | * | * | * |
| **Overall quality score (máximum of eight stars)** | 5 | 6 | 6 | 5 | 4 | 6 | 6 | 7 |

**^*^Note: NOS adapted for cross-sectional studies.**

A study can award a maximum of two stars (representing “yes”) for each item within the sample selection and outcome categories. A maximum of one star can be given for each item within comparability.
